# The Multi Centre Canadian Acellular Dermal Matrix Trial (MCCAT): study protocol for a randomized controlled trial in implant-based breast reconstruction

**DOI:** 10.1186/1745-6215-14-356

**Published:** 2013-10-28

**Authors:** Toni Zhong, Claire Temple-Oberle, Stefan Hofer, Brett Beber, John Semple, Mitchell Brown, Sheina Macadam, Peter Lennox, Tony Panzarella, Colleen McCarthy, Nancy Baxter

**Affiliations:** 1 Division of Plastic & Reconstructive Surgery, University Health Network, Toronto, ON, Canada; 2 Division of Plastic and Reconstructive Surgery, University of Toronto, Toronto, ON, Canada; 3 Plastic Surgery Oncology, Tom Baker Cancer Centre, Alberta Health Services, Calgary, Alberta, Canada; 4 Plastic & Reconstructive Surgery, Women’s College Hospital, Toronto, ON, Canada; 5 Division of Plastic & Reconstructive Surgery, Vancouver General Hospital and the University of British Columbia, Vancouver, BC, Canada; 6 Division of Biostatistics, University Health Network, Toronto, ON, Canada; 7 Plastic & Reconstructive Surgery, Memorial Sloan-Kettering Cancer Center, New York, NY, USA; 8 Department of Surgery, St. Michael’s Hospital, Toronto and the Keenan Research Centre, Toronto, Ontario, Canada

**Keywords:** Breast reconstruction, Implant, Acellular dermal matrix, One-stage, Tissue expander

## Abstract

**Background:**

The two-stage tissue expander/implant (TE/I) reconstruction is currently the gold standard method of implant-based immediate breast reconstruction in North America. Recently, however, there have been numerous case series describing the use of one-stage direct to implant reconstruction with the aid of acellular dermal matrix (ADM). In order to rigorously investigate the novel application of ADM in one-stage implant reconstruction, we are currently conducting a multicentre randomized controlled trial (RCT) designed to evaluate the impact on patient satisfaction and quality of life (QOL) compared to the two-stage TE/I technique.

**Methods/designs:**

The MCCAT study is a multicenter Canadian ADM trial designed as a two-arm parallel superiority trial that will compare ADM-facilitated one-stage implant reconstruction compared to two-stage TE/I reconstruction following skin-sparing mastectomy (SSM) or nipple-sparing mastectomy (NSM) at 2 weeks, 6 months, and 12 months. The source population will be members of the mastectomy cohort with stage T0 to TII disease, proficient in English, over the age of 18 years, and planning to undergo SSM or NSM with immediate implant breast reconstruction. Stratified randomization will maintain a balanced distribution of important prognostic factors (study site and unilateral versus bilateral procedures). The primary outcome is patient satisfaction and QOL as measured by the validated and procedure-specific BREAST-Q. Secondary outcomes include short- and long-term complications, long-term aesthetic outcomes using five standardized photographs graded by three independent blinded observers, and a cost effectiveness analysis.

**Discussion:**

There is tremendous interest in using ADM in implant breast reconstruction, particularly in the setting of one-stage direct to implant reconstruction where it was previously not possible without the intermediary use of a temporary tissue expander (TE). This unique advantage has led many patients and surgeons alike to believe that one-stage ADM-assisted implant reconstruction should be the procedure of choice and should be offered to patients as the first-line treatment. We argue that it is crucial that this technique be scientifically evaluated in terms of patient selection, surgical technique, complications, aesthetic outcomes, cost-effectiveness, and most importantly patient-reported outcomes before it is promoted as the new gold standard in implant-based breast reconstruction.

**Trial registration:**

ClinicalTrials.gov: NCT00956384

## Background

Mastectomy remains a common form of treatment for breast cancer [1,2]. In addition, there has been a ‘rising tide’ in mastectomy utilization that can be attributed to more skin-sparing mastectomies (SSMs) performed concurrently with immediate breast reconstruction. This rise may be attributed to better identification of women at high risk for breast cancer with genetic testing, more refined methods of imaging, and a clearer picture of the late adverse effects of breast irradiation [1-4]. Immediate breast reconstruction has proven to be a safe and beneficial treatment for women diagnosed with early-stage breast cancer, and offers the benefits of improved body image, health-related quality of life (HRQOL), and patient satisfaction [5-12]. For women who have the option of undergoing breast conserving therapy or mastectomy, the selection of SSM with immediate reconstruction is preferred by those who want to avoid radiation and local recurrence, but do not wish to live with a mastectomy defect [13].

**Figure 1:**
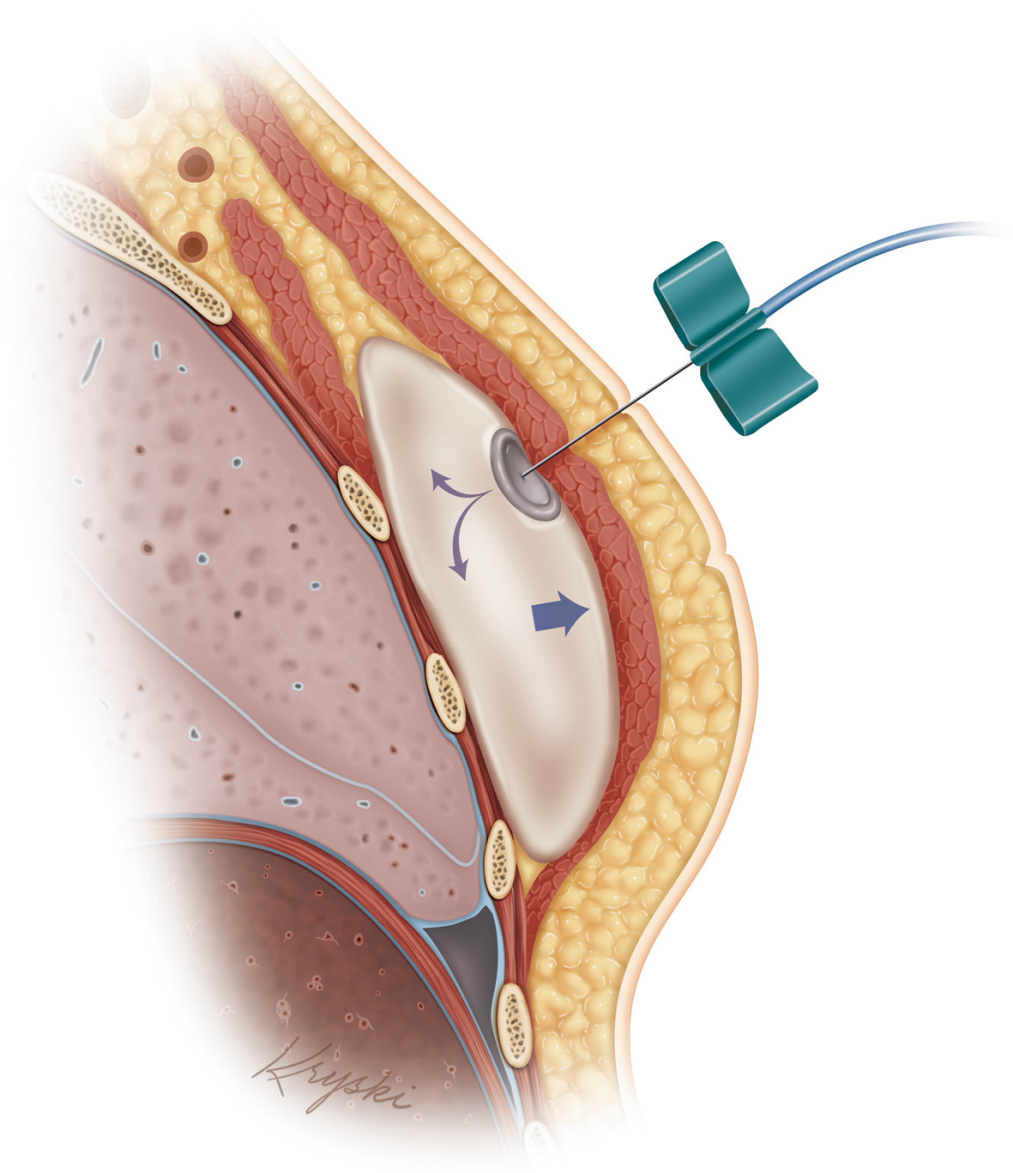
**Schematic drawing of the tissue expansion process.** The location of the tissue expander (TE) is deep to the pectoralis major muscle and is relatively deflated at the initial time of placement, and then gradually inflated following surgery.

In 2007, an estimated 57,000 breast reconstructions were performed to restore mastectomy defects, and of those the majority of cases (34,017) were comprised of the two-stage, tissue expander/implant (TE/I) technique in the USA [14]. The two-stage TE/I reconstruction became the gold standard in implant-based immediate breast reconstruction due to the high complication rates (25% overall explantation rate) associated with the historical, non-ADM assisted method of one-stage implant reconstruction, which placed the full weight of the implant on the vulnerable inferior mastectomy flap [15]. In the first stage immediately following mastectomy, a tissue expander (TE) is temporarily placed under a complete musculofascial cover made up of pectoralis muscle and rectus fascia [16]. Because the musculofascial cover is non-distensible and excessive pressure on the vulnerable mastectomy flap should be avoided, a partially filled temporary TE is placed immediately following mastectomy. Postoperatively, serial expansion is performed weekly in the clinic and exchange of the temporary TE for a permanent implant occurs at a subsequent operation (Figure 1).

**Figure 2:**
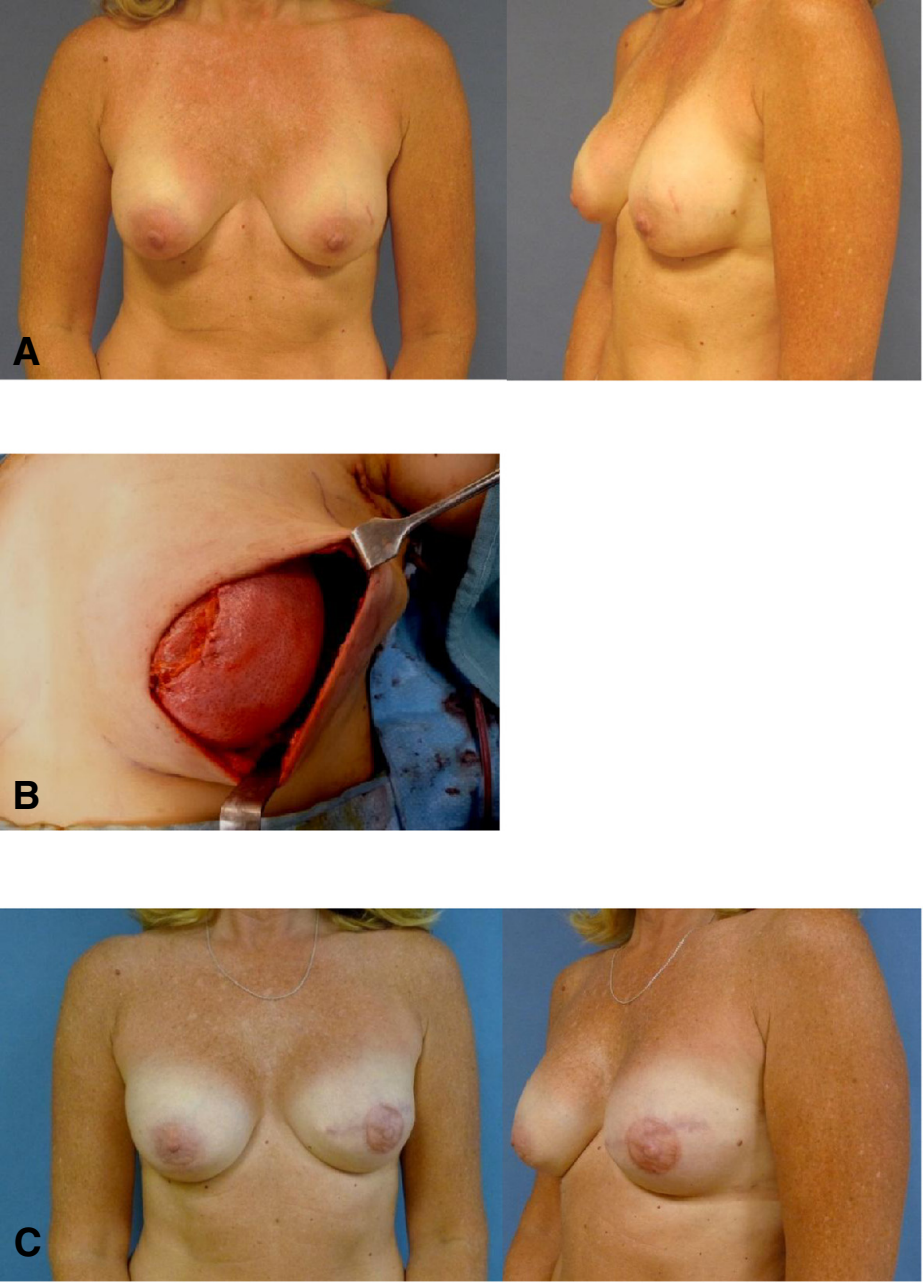
**Preoperative, intraoperative, and postoperative photos of the one-stage acellular dermal matrix (ADM)-assisted implant reconstruction following left skin-sparing mastectomy (SSM). (A)** Preoperative anterior and oblique views, prior to left mastectomy and reconstruction. **(B)** Intraoperative view of ADM acting as an ‘internal hammock’ and insertion of a full-sized permanent implant. **(C)** Postoperative views, following left SSM, one-stage ADM reconstruction and nipple areolar complex reconstruction. ADM, acellular dermal matrix; SSM, skin-sparing mastectomy.

To address the inconvenience associated with the two surgeries in the TE/I method, while avoiding the high complication rates associated with the traditional one-stage technique, a number of newer one-stage implant reconstruction methods have been described [17,18]. One useful technique that can allow the breast mound to be created in a single stage using a permanent full-sized breast implant is with the use of ADM [19-21]. ADM is an immunologically inert dermal material derived from cadaveric human skin tissue that evades host rejection and is safe for use in the human body. Immediately following mastectomy, ADM is used to extend the musculofascial cover and in turn creates a large enough space to accommodate a fully inflated implant. By acting as an ‘internal hammock’ to support the implant, it minimizes the tension that is exerted on the vulnerable mastectomy flap (Figure 2).

Lastly, revascularization from the surrounding tissues and the ability of ADM to be incorporated into the host tissues prevent extrusion of the implant [19-21]. It has been recently described in numerous case reports that ADM can successfully allow one-stage implant reconstruction to take place without the use of TEs [20-25]. Salzberg *et al*. reported the largest series of immediate one-stage breast reconstruction using ADM (AlloDerm, LifeCell, Branchburg, NJ, USA) in 260 patients (466 breasts) over an 8-year period [23]. After a mean follow-up of 29 months, the overall complication rate was 3.9%, which is comparable to complication rates described for two-stage TE/I reconstructions [26,27]. On the other hand, however, some reports have suggested that ADM is associated with higher rates of infection and seroma formation [24,25], and there is a paucity of research on its long-term aesthetic outcomes.

In order to rigorously investigate the novel application of ADM in one-stage implant reconstruction, we are currently conducting a multicentre randomized controlled trial (RCT) designed to evaluate the impact on patient satisfaction and quality of life (QOL) compared to the two-stage TE/I technique, the gold standard in implant-based breast reconstruction. We hypothesize that the ability to convert a conventional two-stage procedure to only one step would improve patient satisfaction, decrease the morbidity associated with tissue expansion and two surgeries, and justify the cost of this biomaterial incurred by the patient, hospital, or healthcare system.

### Primary aim of study

1) Compare the mean change in the patient satisfaction and QOL scores between one-stage ADM-assisted and two-stage TE/I reconstruction without ADM at 12 months following reconstruction using the validated BREAST-Q reconstruction module [28].

### Secondary aims of study

1) Compare the change in the BREAST-Q global score over time (2 weeks, 6 months, 12 months) following reconstruction between the two implant reconstruction methods.

2) Compare the short- and long-term operative complication rates.

3) Compare overall aesthetic outcomes using five standardized photographs graded by three independent blinded observers using the three-point, five-item, Lowery breast aesthetic score at 1-year [29,30].

4) Perform a cost-effectiveness analysis of the two different methods of implant reconstruction using person-level cost and effect data.

## Methods/design

**Figure 3:**
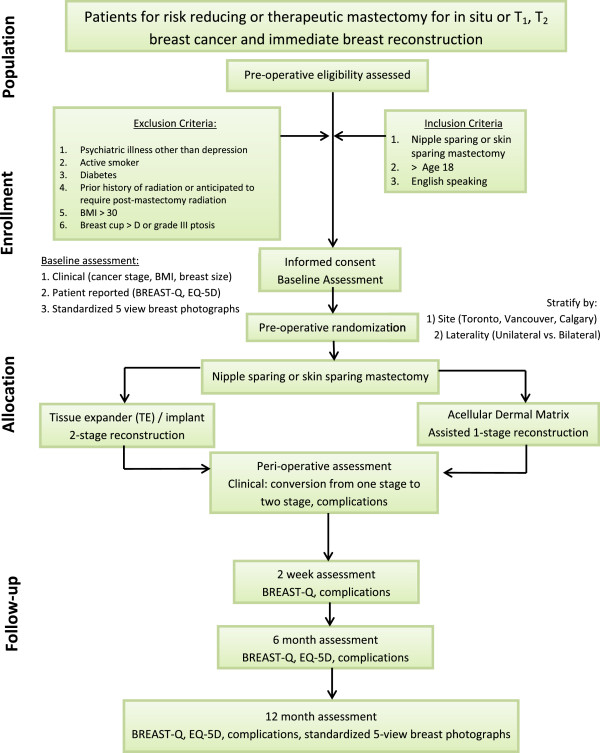
Study design flow chart.

The MCCAT study is a multicentre randomized controlled two-arm parallel superiority surgical trial that will compare ADM-facilitated one-stage implant reconstruction compared to two-stage TE/I reconstruction following SSM or nipple-sparing mastectomy (NSM) at 2 weeks, 6 months, and 12 months. Stratified randomization will maintain a balanced distribution of important prognostic factors across interventions. Stratification variables will include *study site* (three strata: Toronto hospitals, ON, Canada; Vancouver General Hospital, Vancouver, BC, Canada; Tom Baker Cancer Centre, Calgary, AB, Canada) and *laterality of surgery* (two strata: unilateral versus bilateral) (Figure 3). This study was approved by the research ethics board of each institution, including: Toronto hospitals, University Health Network Research Ethics Board (ethics ID 09-0267-A), Women’s College Hospital Research Ethics Board (ethics ID 2009-0018-B); Vancouver General Hospital, University of British Columbia Clinical Research Ethics Board (ethics ID H09-01898); and Tom Baker Cancer Centre, University of Calgary Conjoint Health Research Ethics Board (ethics ID E-24812). The MCCAT is an investigator-initiated trial, sponsored by the institution of the coordinating site (University Health Network).

### Participants

The source population will be women planning to undergo risk-reducing mastectomy for *BRCA* gene positivity or therapeutic mastectomy for stage T0 to TII breast cancer with implant-based immediate breast reconstruction at one of the four study sites. Participant eligibility will first be determined by the investigating surgeon at the preoperative consultation. Patients will be excluded if they have a documented psychiatric history except depression and anxiety, prior history of breast irradiation or anticipated to need postoperative irradiation, or are active smokers, body mass index (BMI) greater than 30, or greater than D cup breast size or grade III breast ptosis.

### Recruitment and enrollment

New patients considering implant-based immediate breast reconstruction will be approached for the study during the initial surgical consultation by the investigating surgeon. Furthermore, information regarding the trial is available online through a trial-specific website, since many patients choose to educate themselves on their options prior to initial consultation with the plastic surgeon. During the consultation, the investigating surgeon will first determine if the patient is eligible to join the trial. If eligibility has been confirmed, the surgeon will explain the trial and the two surgical procedures under investigation, including the benefits, risks, and complications of each option. If the patient is interested in the trial, then the study coordinator will meet with the patient and provide a detailed explanation of all aspects of the research study. Realistic photos of the results of both procedures will be presented to the patients, as well as examples of the implants, ADM, and TEs. Additionally, if desired, the patient may be referred to speak with patient volunteers who have previously undergone the same procedures. After adequate consideration of their options, if the patient agrees to join the trial, then the study coordinator will conduct the informed consent process.

### Randomization allocation and blinding

Once informed consent and baseline questionnaires have been obtained, the study coordinator will obtain the patient’s randomization arm from opaque, sealed envelopes in sequence to determine the participant’s randomized treatment allocation. Blocked randomization will be incorporated into each of the six individual strata (site: three strata; breast laterality: two strata) in fixed blocks of eight. The randomization allocation list will be developed by a statistician using PROC PLAN in SAS (SAS Institute Inc, Cary, NC, USA). Stratified randomization will maintain a balanced distribution of important prognostic factors across interventions and control for: 1) confounding related to trial site; and 2) possible unequal proportions of unilateral versus bilateral procedures. Due to the non-pharmacologic nature of the intervention, it is not possible to use a placebo and patients will be informed of their assigned treatment weeks prior to their scheduled surgery. In addition, the plastic surgeon performing the procedure will not be blinded. To reduce the impact of participant ascertainment bias, study participants will be told that the aim of the study is to compare different methods of breast reconstruction without identifying the experimental intervention.

### Surgical interventions

In both procedures, the SSM or NSM will be performed using incisions designed by the plastic surgeon. In most cases, an ellipse will be designed around the nipple areolar complex for the skin-sparing approach, and either a periareolar or inframammary fold incision will be used for the nipple-sparing approach. Following completion of the mastectomy, the plastic surgeon will first create the implant/TE pocket by elevating the pectoralis major muscle to the preoperatively marked footprint of the breast pocket. In the *experimental group*, the ADM will be used to create the inferolateral aspect of the pocket. An anatomic cohesive silicone gel (Style 410, Allergan, Santa Barbara, CA, USA, or Mentor CPG 323, Santa Barbara, CA, USA) will be placed in the pocket and closure of the pocket will use an absorbable suture. In Group B, the *control group*, in addition to elevating the pectoralis major muscle, serratus anterior muscle/fascia laterally and rectus fascia inferiorly, a partially saline filled anatomic-shaped TE (Allergan) will be placed beneath the musculofascial pocket and closed using an absorbable suture. In both procedures, one drain per breast will be placed deep to the mastectomy skin flap along the inframammary recess, and an additional drain per breast will be placed deep to the ADM in the experimental arm. All patients will receive standard perioperative antibiotics. All participating surgeons are staff surgeons who have experience of both the one-stage and two-stage procedures, and will perform the assigned surgery that their patient is randomized to receive. Patients will be informed prior to surgery that there is a risk that the one-stage procedure will be converted to a two-stage procedure (insertion of TE with ADM) if the mastectomy flap is deemed too thin to support a full-sized permanent implant intraoperatively.

#### Interventional agent

ADM (AlloDerm) derived from cadaveric human skin tissue is supplied by American Association of Tissue Banks (AATB)-compliant tissue bank, and adheres to the standards of the AATB and the Food and Drug Administration’s guidelines. AlloDerm is a human-derived ADM approved by Health Canada. The authors have no conflicts of interest with Allergan or LifeCell. For postoperative treatment in the control group, tissue expansion will take place on a weekly or biweekly basis until the TE is filled to the patient’s desired volume or matches the patient’s contralateral side. All patients in the control arm will undergo a second exchange procedure, at which time the TE is removed and a permanent implant is placed using an anatomic cohesive silicone gel implant at 3 (±3) months.

### Primary outcomes

To compare the change in patient satisfaction and QOL between the two surgical interventions from baseline to 12 months following completion of reconstruction, we will use the breast reconstruction module of the BREAST-Q. A patient-reported outcome was selected as the primary outcome measure as the main goal of breast reconstruction surgery is to improve patient QOL [28,31-34]. The 12-month time-point was selected because there is evidence that QOL evolves dynamically in the first year following breast cancer surgery and plateaus at 1-year [35,36]. The BREAST-Q reconstruction module is a patient-reported outcome measure that was developed to assess HRQOL and patient satisfaction following breast reconstruction [28,31-34]. It contains six subscales that measure well-being and satisfaction before and after reconstruction. A global score that is a summation of satisfaction and QOL can be transformed on a scale from 0 to 100 using the RUMM software (RUMM Laboratory, Perth, Australia). The measure has excellent reliability, with high internal consistency (Cronbach’s alpha from 0.88 to 0.96) and test-retest reliability (intraclass correlation coefficient (ICC) 0.85 to 0.98) for the BREAST-Q subscales [28].

### Secondary outcomes

*Satisfaction and QOL changes over time* will be measured at discrete time-points of 2 weeks, 6 months, and 12 months following completion of reconstruction, taking into account time effect and time-group interaction as shown on Table 1.

**Table 1:** Timetable of interventions and outcome measurements

	**Baseline**		**Week 2**	**Week 3**	**Week 4**	**Week 5**		**2**	**6**	**12**
	**(preoperative)**							**weeks**	**months**	**months**
Group A visits to clinic	x	*Mastectomy and reconstruction*	x		x			x	x	x
Group B visits to clinic	x		Expansion	Expansion	Expansion	Expansion	*Expander exchange*	x	x	x
BREAST-Q	x							x	x	x
EQ-5D	x								x	x
Complications			x	x	x	x		x	x	x
Aesthetic assessment	x									x

*Short and long-term complication rates* will be documented and collected prospectively on intraoperative and postoperative assessment forms. Short-term complications are those that occur intraoperatively or within the first 2 months of surgery. Surgical outcomes and complications will be compared between the two procedures as outlined on Table 2.

**Table 2:** Intraoperative, early and late postoperative surgical outcomes, and complication data collection

**Intraoperative**	
**Common intraoperative outcomes**	
Admission status (admitted/not admitted)	
Need for completion axillary lymph node dissection (yes/no)	
Mastectomy flap deemed to have significant ischemia (yes/no)	
Significant mastectomy flap thickness asymmetry between two sides (yes/no)	
Mastectomy weight (right breast (g)/left breast (g))	
Mastectomy flap thickness (significant dermal exposure/patches of dermis exposed/thin flap without dermis exposed/normal)	
Skin incision (horizontal ellipse/vertical ellipse/oblique ellipse/nipple-sparing with inframammary fold incision/nipple-sparing with lateral extension)	
Sentinel lymph node biopsy (yes/no, left- and right-side)	
Number of drains	
Tension with skin closure (present/absent)	
*First stage of the two-stage*	*Direct to implant with ADM*
TE style	Excess skin excision precluding one-stage implant reconstruction (yes/no)
Initial fill volume (cc)	Size of ADM (cm^2^)
	Implant style
	Implant volume (g)
	Implant surface and shape (round/anatomic or textured/smooth)
	If TE inserted in the place of an implant: reason (implants too large/too small/flap necrosis suspected)
*Second stage surgery (exchange to implant)*	
Total TE fill volume (cc)	
Capsulectomy (total/subtotal/none)	
Capsulotomy (yes/no)	
Capsulorrhapy sutures (yes/no)	
Implant size and style	
Implant surface and shape (round/anatomic or textured/smooth)	
**Within first 2 months of surgery**	
**Common outcomes**	
Signs of seroma (yes/no, if yes, amount of fluid drained (cc))	
Mastectomy flap viability (100%, 95 to 100%, 80 to 95%, 60 to 80%, <60%)	
Need for debridement of necrosis (yes/no, if yes, debrided (cm^2^))	
Signs of infection (yes/no, if yes, name and dosage of antibiotics prescribed)	
Signs of incisional dehiscence (yes/no)	
Other complications/additional comments	
Number of days until drains removed	
*Two-stage*	*One-stage with ADM*
Need for deflation of TE (yes/no)	Signs of implant malposition (yes/no)
Signs of TE malposition (yes/no)	Signs of implant exposure (yes/no)
Signs of TE exposure (yes/no)	Signs of ADM exposure (yes/no)
Expansion each visit (cc)	
**Long-term complications common to both procedures**	
Signs of implant malposition (yes/no)	
Signs of implant exposure (yes/no)	
Signs of implant rippling (yes/no, if yes, superior/medial)	
Symmetry (overall) (poor/fair/good/outstanding/excellent)	
Symmetry of inframammary fold (poor/fair/good/outstanding/excellent)	
Hypertrophic or keloid scar present (yes/no, assessed for each breast)	
Aesthetics (poor/fair/good/outstanding/excellent, assessed for each breast)	
Capsular contracture (none/II barely visible, palpable/III visible, palpable/IV severe, painful, assessed for each breast)	

ADM, acellular dermal matrix; TE, tissue expander.

*Long-term aesthetic result* will be evaluated by a panel of three independent blinded observers using the three-point, five-item breast aesthetic score on standardized five-view photographic documentation at 12-month follow-up [29,30].

*Cost-effectiveness analysis* will be simultaneously analyzed by collecting person-level cost and effect data. The cost data will come from hospital cost accounting systems and patient reports. The patient outcome data will be collected by the BREAST-Q and the frequently used generic EQ-5D (EuroQol Group, Rotterdam, The Netherlands) utility measure. The primary measure of effectiveness will be the BREAST-Q score and the secondary measure will be the quality-adjusted life year (QALY) as measured by the EQ-5D.

### Data collection

Adherence to the protocol can be enhanced when postoperative outcomes are obtained during routine patient follow-up with their plastic surgeons. Thus, the primary and secondary patient-reported outcome measures will be administered to the patient at the follow-up clinic visits by the study coordinator. In addition, the investigating surgeons will complete the assessment of short- and long-term complications and surgical outcomes at each postoperative follow-up visit using standardized data collection forms (Table 2). For the long-term assessment, photographs will be taken by the study coordinator or the surgeon at the 12-month follow-up visit. The schedule of data collection during follow-up is shown in Table 1.

### Data analysis plan

Given the stratified design, incorporating study site, status of breast cancer, and a continuous primary outcome, differences in the two treatments will be tested using an analysis of variance model. Baseline BREAST-Q measurements and other potentially confounding variables (BMI, age, presence of chemotherapy or hormonal therapy) that have been not balanced by randomization will be adjusted for in the model. For secondary analyses, we will also investigate the possibly variable effects of the treatment group differences across multiple time-points, namely 2 weeks, 6 months, and 12 months postoperatively. A linear mixed regression model will be used where correlation between the three time-points within a patient are appropriately accounted for in the statistical inferences drawn. Potentially confounding variables will be adjusted for in the model fit. In particular, we will test the interaction between treatment and time. The occurrence of short- as well as long-term complications will be compared between the surgical intervention groups using Fisher’s exact test. Although confounding is not expected due to randomization of patients to intervention groups, any confounding factors will be adjusted using multivariable logistic regression models. The logistic regression will also provide knowledge of the relationships and strengths among the variables. The median breast aesthetic score (I to V) for each group will be compared using the Mann–Whitney *U* test at 12-month follow-up. All analyses will follow the intention-to-treat principle. Analyses will be run primarily using SAS version 9.2. The competing risk analyses will be run using the cmprsk package in R version 2.11.1 (Institute for Statistics and Mathematics, Vienna University of Economics and Business, Vienna, Austria).

### Sample size

The primary endpoint evaluated will be the average change in patient-reported satisfaction and QOL summary score measured using the validated BREAST-Q from baseline compared to 12 months following reconstruction between the two techniques. We consider clinically relevant change in the QOL as a difference that exceeds half a standard deviation of the baseline value. We wish to detect a minimally clinically important difference of 10 as statistically significant at the 0.05 level (two-sided), with a power equal to 0.85. Assuming a standard deviation of 20 (through field testing of BREAST-Q) and one interim analysis using an O’Brien-Fleming spending function stopping boundary, a total of 73 evaluable patients per treatment group is needed. The sample size calculation was run using PASS 2008 (NCSS, Kaysville, UT, USA). Our sample size calculation takes into account stratification, which increases our power to detect differences in the primary outcome between the two groups, and since our primary aim is not to conduct formal hypothesis testing on the effects of stratification on the outcome, the sample size calculation reflects a conservative estimate [37]. Assuming a 1:1 allocation ratio, 146 patients in total are required. If we further assume a 10% loss to follow-up, we would need to accrue 146/(1 – 0.1) = 162 patients. If the result of the interim analysis *P* value is less than 0.003 then we would consider stopping the study early. The specific boundaries of the stopping rule adjust for multiple looks at the data, while ensuring an overall type I error of 0.05. The interim analysis will be conducted by the Data and Safety Monitoring Board (DSMB) at the Clinical Trials Support Unit at Princess Margaret Hospital, Toronto, ON, Canada. One interim review by the DSMB will be setup after half of the patients (n = 81) have completed 1-year follow-up and are evaluable using the O’Brien-Fleming spending function stopping boundary.

## Discussion

Since the feasibility of using ADM in both one-stage and two-stage prosthetic breast reconstruction was first established in the literature with several case series, ADM has ‘exploded into the marketplace and has been integrated into the practices of many plastic surgeons who perform prosthetic breast reconstruction’ [20-22,38-43]. In a 2010 survey of US plastic surgeons, over half reported frequent use of ADM as an adjunct to implant breast reconstruction [44]. When used as an inferolateral hammock, ADM is considered to enhance the breast aesthetic by better defining the IMF, allowing more lower pole fullness and reducing the severity of capsular contraction [20].

Despite the widespread adoption of ADMs in the setting of implant reconstruction, not all evidence on ADM has been favorable. This may reflect the early learning curve in patient selection and technique with the use of a new surgical method, or it may be real. A recent retrospective review of 337 two-stage TE/I reconstructions with and without ADM found no significant difference between the two groups in terms of capsular contracture and mechanical shift in the multivariate analysis [42]. Since using ADM allows for significantly higher initial TE fill volume [40], some authors consider that the most important application of ADM is in the setting of one-stage direct to implant reconstruction or inadequate local muscle coverage of the implant [20-22,38,39,41]. However, in the only RCT that examined the use of ADM in a two-stage TE/I reconstruction, the authors found no difference in postoperative pain or the rate of expansion between TE/I alone compared to TE/I using ADM as an inferolateral hammock.

Despite the mixed evidence on ADM, there is strong interest in using ADM in implant breast reconstruction particularly in the setting of one-stage direct to implant reconstruction where it was previously not possible with the use of implants alone, and always required an intermediate stage with a temporary partially-inflated breast expander. This unique advantage has led many patients and surgeons alike to believe that one-stage ADM-assisted implant reconstruction should be the procedure of choice, and should be offered to patients as the first-line treatment. Although we acknowledge and share the sentiments on the advantages of ADM-assisted reconstruction, given its cost and higher risk of infection and seroma, we maintain our firm position that this technique should be scientifically evaluated in terms of patient selection, surgical technique, complications, aesthetic outcomes, cost-effectiveness, and most importantly patient-reported satisfaction and QOL outcomes before it is put forth as the new gold standard in implant-based breast reconstruction. The difficulty of undertaking such an evaluation, however, exists on many levels and can be succinctly summarized in Buxton’s law: ‘It is always too early [for rigorous evaluation] until, unfortunately, it’s suddenly too late’ [45]. The use of ADM-assisted one-stage direct to implant reconstruction may become another example of the *ad hoc* nature in which a new surgical intervention is introduced into practice without first undergoing a rigorous evaluation beforehand. Therefore, the MCCAT study has at present a singular and critical opportunity to use level I evidence to evaluate the efficacy of converting a traditional two-stage TE/I method to a single stage procedure with the use of ADM on patient-reported outcomes, complications, aesthetic results, and cost-effectiveness.

### Risks and benefits

Most of the risks in this study are common minor and major surgical complications shared by both the one- and two-stage implant breast reconstruction procedures (Table 3). The risk that is unique to the ADM group includes the rare possibility of pathogen or disease transmission from the processed human cadaveric donor tissue. However, there have not been any case reports of disease or infection transmitted by human ADM to date. A final important risk is that in about 5% of the cases where subjects are randomized to the ADM-assisted one-stage arm, if the mastectomy flap thickness is found to be too thin or avascular to provide healthy cover for the ADM/implant construct, then the technique will be converted to the traditional TE insertion technique. The most important benefit that can be derived from this study is the possibility of having a breast mound completely reconstructed in a single stage at the same time as the mastectomy that was previously deemed unsafe without the use of ADM.

**Table 3:** Surgical risks to participants

**Surgical complications common to both surgical methods**	
**Major complications**	**Minor complications**
Implant or TE infection requiring removal of the prosthesis (3%, 80% reversible)	Implant or TE infection requiring only antibiotics (3% not severe, 100% reversible)
Mastectomy skin flap problems resulting in removal of implant or TE (2%, 80% reversible)	Mastectomy skin flap problems requiring only conservative treatment and minor debridement in clinic (2% not severe, 100% reversible)
Implant movement requiring additional surgery (3%, 80% reversible)	Visible implant rippling, shape deformity, or poor alignment not requiring correctional surgery (10 to 20%)
Implant rippling, shape deformity, or poor alignment requiring surgery to correct it (10% not reversible)	
	Seroma or hematoma (1 to 2% not severe, 100% reversible)
	Long-term capsular contracture formation (25% develop grades III to IV in 10 years, not reversible)
**Possible complications present in the two-stage TE/I method**	**Possible complications present in the ADM one-stage method**
All need a second surgery for TE exchanged to implant under another general anesthetic	Hypothetical risk of pathogen or disease transmission from the processed human cadaveric donor tissue
All need at least one postoperative TE inflation, on average three inflations that are performed either weekly or biweekly	One-stage method may not be possible for patients who wish to have larger breast sizes postoperative than they had preoperative
All need to wait approximately 3 (±1) months following completion of the TE expansion process prior to the second surgery	Need for two drains rather than one drain per breast
May be discomfort associated with TE inflation	Some reports of increased seroma formation with the use of ADM
Saline leak from the TE may occur and can result in an additional surgery	May be more pain postoperatively with suturing of the ADM down to the chest wall along the inframammary fold
TE may be malpositioned or migrate postoperatively requiring revision surgery prior to TE exchange	There may be more asymmetry between the two sides since there is only surgery to reconstruct the breast mound (versus two opportunities for symmetry correction in the two-stage)
May be additional complications resulting from two surgeries	Less control with the patients selecting the volume of their reconstructed breasts compared to the two-stage TE/I group
May be higher risk of hematoma formation from the need to elevate pectoralis major, serratus major, and rectus fascia	
May be more surgical pain associated from the need to elevate pectoralis major, serratus major, and rectus fascia	
All need a second surgery for TE exchanged to implant under another general anesthetic	

ADM, acellular dermal matrix; TE, tissue expander; TE/I, tissue expander/implant.

### Ethical considerations

Clinical equipoise exists in this trial since no prior studies have compared patient-reported outcomes or risks/complications following the two types of implant-based breast reconstruction. Institutional Research Ethics Board (REB) approval has been obtained for all the study sites along with the data share agreements with the primary study site. Research staff will also ensure that protocol subjects receive Notice of Privacy Practices. Any serious adverse events will be reported to the REB as soon as possible but no later than 5 calendar days.

### Limitations and strengths

Blinding of participants and surgeons is impossible given the intervention. This may bias the primary outcome since QOL may seem a subjective endpoint; however, the BREAST-Q is a validated, reliable assessment of QOL that should provide a robust measure of satisfaction. The assessment of the overall aesthetic outcome, however, uses independent blinded judges and therefore reduces the bias when evaluating this secondary outcome. Secondly, although it would have been preferable to stratify important baseline covariates such as breast cancer stage (prophylactic versus *in situ* versus invasive breast cancer), it would have added too many strata relative to our sample size. In addition, although stratification based on whether the patient will require adjuvant chemo- or hormonal therapy would have been desirable, this information is not generally available at the time of randomization, nor is it in ‘real life’, as it only becomes known with the final pathologic evaluation of the mastectomy and sentinel lymph node. Therefore, our approach will be to adjust these confounders in the analysis. The 1-year duration for follow-up does not take into account revision surgeries that occur later than 1 year, therefore interpretation of the results should also be limited to this duration. The greatest strength of this study is the use of the randomized controlled design as the most stringent study design to elucidate a true cause-effect relationship between our intervention (one-stage ADM reconstruction) and effect (improved patient outcomes) by balancing both known and unknown variables in a relatively mixed mastectomy population.

## Trial status

### Trial inception

The MCCAT study was initially designed as a multicentre trial that involved only two centres in Toronto, ON, Canada, and began enrollment in October 2009. After 1.5 years, the third centre, Vancouver General Hospital, BC, Canada, was recruited in April 2011, followed by the fourth centre in Calgary, AB, Canada, in January 2013 to increase recruitment and generalizability of results.

### Eligible patients

At the date of manuscript preparation, a total of 149 patients have been deemed eligible and 121 patients have been enrolled into the trial.

### Operated patients

In addition, 106 patients have undergone their allocated intervention (mastectomy surgery with either TE insertion or one-stage direct to implant using ADM; seven patients are awaiting surgery, four patients withdrew from the study, one patient was deemed ineligible, three patients did not receive allocated intervention). To date, 63 patients have completed the trial and are evaluable for the primary outcome.

### Protocol amendment

The protocol was amended in October 2011 to include an additional time-point for completing the BREAST-Q instrument in the control group at 6 months following mastectomy and insertion of TE after the first stage. The research team considered that although patients were still in the mid-process of reconstruction during expansion, answering the BREAST-Q would provide a better understanding of the impact of reconstruction on their interim QOL.

## Abbreviations

AATB: American association of tissue banks; ADM: Acellular dermal matrix; BMI: Body mass index; DSMB: Data and safety monitoring board; HRQOL: Health-related quality of life; ICC: Intraclass correlation coefficient; MCCAT: Multi centre Canadian acellular dermal matrix trial; NSM: Nipple-sparing mastectomy; QALY: Quality-adjusted life year; QOL: Quality of life; RCT: Randomized controlled trial; REB: Research ethics board; SSM: Skin-sparing mastectomy; TE: Tissue expander; TE/I: Tissue expander/implant.

## Competing interests

TZ, MB, SM, and PL have been paid speakers for LifeCell in the past 5 years.

## Authors’ contributions

TZ is the primary grant holder of this study. As the primary author, TZ designed the protocol, and is currently leading the study, screening, recruiting patients, operating, and conducting follow-up assessments. TZ will oversee data analysis and interpretation, as well as write the manuscript. CTO is screening, recruiting, operating on patients, conducting follow-up assessments, and will contribute to data interpretation and manuscript writing. SH, BB, JS, and MB designed the study, are screening and recruiting eligible patients, operating on patients, conducting follow-up assessments, and will contribute to data interpretation and manuscript writing. SM and PL are screening and recruiting eligible patients, operating on patients, conducting follow-up assessments, and will contribute to data interpretation and manuscript writing. CM helped to design the study. TP provided statistical support. NB is a grant holder, helped design the study, is contributing expertise to study implementation, and will also contribute to data analysis and manuscript writing. All authors read and approved the final manuscript.
