# Erratum to: The Multi Centre Canadian Acellular Dermal Matrix Trial (MCCAT): study protocol for a randomized controlled trial in implant-based breast reconstruction

**DOI:** 10.1186/s13063-016-1179-6

**Published:** 2016-01-20

**Authors:** Toni Zhong, Claire Temple-Oberle, Stefan O. P. Hofer, Brett Beber, John Semple, Mitchell Brown, Sheina Macadam, Peter Lennox, Tony Panzarella, Colleen McCarthy, Nancy Baxter, For the MCCAT Study Group

**Affiliations:** Division of Plastic and Reconstructive Surgery, University Health Network, Toronto, ON Canada; Plastic Surgery Oncology, Tom Baker Cancer Centre, Alberta Health Services, Calgary, AB Canada; Division of Plastic and Reconstructive Surgery, Vancouver General Hospital and University of British Columbia, Vancouver, BC Canada; Division of Biostatistics, University Health Network, Toronto, ON Canada; Plastic and Reconstructive Surgery, Memorial Sloan-Kettering Cancer Center, New York, NY USA; Department of Surgery, St Michael’s Hospital, Toronto, and Keenan Research Centre, Toronto, ON Canada

## Erratum

After publication of this work [1], we noted that we failed to include the middle initials of the author, Stefan O.P. Hofer. The full list of authors has now been updated. We are publishing this erratum to update the author list, which is as follows:

Toni Zhong, Claire Temple-Oberle, Stefan O.P. Hofer, Brett Beber, John Semple, Mitchell Brown, Sheina Macadam, Peter Lennox, Tony Panzarella, Colleen McCarthy and Nancy Baxter

## References

[1] Zhong T, Temple-Oberle C, Hofer S, Beber B, Semple J, Brown M, et al. The Multi Centre Canadian Acellular Dermal Matrix Trial (MCCAT): study protocol for a randomized controlled trial in implant-based breast reconstruction. Trials. 2013;14:356 10.1186/1745-6215-14-356 PMC3842809 24165392

